# Racial and socioeconomic disparities from time of diagnosis to treatment for small renal masses

**DOI:** 10.1002/bco2.70115

**Published:** 2026-02-03

**Authors:** Lila G. McGrath, Hailey W. Holck, Anthony J. Teixeira, Mallie C. Roley, Saeed Dupree, Ferdous Ahmed, Kris E. Gaston, Justin T. Matulay, Stephen B. Riggs, Peter E. Clark, Ornob P. Roy

**Affiliations:** ^1^ School of Medicine Wake Forest University Winston‐Salem NC USA; ^2^ Medical School UT Southwestern Dallas TX USA; ^3^ School of Medicine Vanderbilt University Nashville TN USA; ^4^ Department of Urology Atrium Health/Levine Cancer Institute Charlotte NC USA; ^5^ Johnson C. Smith University Charlotte NC USA; ^6^ Department of Biostatistics and Data Sciences Atrium Health/Levine Cancer Institute Charlotte NC USA

**Keywords:** healthcare disparities, renal cell carcinoma, small renal masses, socioeconomic status, time to treatment

## Abstract

**Objectives:**

To investigate how patient‐specific factors, including race and socioeconomic status, impact time to treatment initiation (TTI) for patients with small renal masses (SRMs).

**Materials and Methods:**

We retrospectively reviewed 275 patients with SRMs ≤ 4 cm at Atrium Health Carolinas Medical Center who underwent treatment for their renal mass. TTI was defined by the time between office visit (TTI‐OV) or between initial imaging (TTI‐Imaging) and procedure date. Statistical analysis was employed to determine patient‐specific factors associated with TTI.

**Results:**

We found that TTI was significantly associated with race as Black patients experienced longer TTI than non‐Hispanic White patients (OV: HR = 0.637, 95% CI [0.479–0.848], p = 0.0048; Imaging: HR = 0.541, 95% CI [0.402–0.727], p = 0.0002). TTI, however, was not significantly associated with socioeconomic status as defined by Area Deprivation Index, income or insurance status. TTI‐OV was also significantly associated with procedure year, and TTI‐Imaging was associated with procedure year, Charlson Comorbidity Index (CCI) and tumour size when first seen on imaging. On multivariable analysis, TTI‐Imaging was not independently associated with race (p = 0.1775), suggesting procedure year, CCI and tumour size are more significant predictors of TTI.

**Conclusion:**

Black patients experienced a treatment delay from initial clinical presentation to procedure, but treatment delays from initial imaging identification to procedure may be tied more strongly to clinical factors.

## 1 INTRODUCTION

Renal cell carcinoma (RCC) is the most common type of kidney cancer, accounting for approximately 90% of cases.^1^ The incidence of RCC has increased over the past few decades, in part due to earlier diagnosis from the increased use of radiological imaging techniques.^2^ Small renal masses (SRMs), which measure 4 cm or less, account for 48–66% of RCC diagnoses. Twenty percent of SRMs are benign, 55–60% are indolent RCCs and 20–25% have potentially aggressive features. Their malignant potential highlights the importance of effective management with active surveillance, surgical intervention and/or ablation, each of which has strengths and weaknesses as management strategies.^3^ Previous research has identified both racial and socioeconomic disparities in the presentation and treatment of patients with RCC. Black patients are 23% more likely to receive non‐surgical treatment compared to other groups.^4^ When Black patients do undergo surgery, they are less likely to receive partial nephrectomy.^4^, ^5^ Additionally, living in higher‐income neighbourhoods has been associated with lower odds of no treatment and lower overall mortality in both Black and White RCC patients.^5^

Time to treatment initiation (TTI) disparities are an underassessed health inequity that can result in longer diagnosis‐to‐treatment times for disadvantaged patients. Current research into the association between TTI and cancer outcomes is conflicting.^6^ While some studies report a lack of association, some data indicate that longer TTI may be associated with worse outcomes for cancer patients.^6^, ^7^ Nonetheless, meta‐analysis suggests that expedited diagnosis and treatment timelines are likely to benefit most cancer patients, albeit to varying degrees.^6^ These time disparities exist across racial groups in different cancer subspecialties. Black breast cancer patients receive late adjuvant hormonal therapy and surgery at higher rates compared to White patients.^8^ In urology, Black prostate cancer patients are more likely to experience longer wait times between diagnosis and treatment compared to White patients, even after accounting for age, marital status, annual household income, level of education and health insurance status.^9^

Patient clinical characteristics may also impact TTI. Colorectal cancer patients with greater comorbidities are less likely to receive adjuvant chemotherapy and surgical treatment.^10^ Comorbidity, therefore, presents a barrier to treatment as additional medical issues must be considered in treatment plans. Additionally, the clinical presentation of the tumour can alter treatment timelines. Clinical tumour size >3 cm is predictive of metastasis in RCC patients, necessitating expedited treatment.^11^ Here, we build upon the growing body of literature on TTI disparities to specifically investigate time disparities in patients with SRMs. We evaluate the prognostic value of race and ethnicity on the length of time from initial diagnosis to treatment for SRMs, in addition to the impact of TTI on survival. We also evaluate additional patient factors that could be associated with treatment delay, such as socioeconomic status (using Area Deprivation Index [ADI] data and census income), insurance status, comorbidity, mass size and year of clinic presentation.

## 2 MATERIALS AND METHODS

### 2.1 Data sources

This study was a retrospective review of an IRB‐approved prospectively managed Small Kidney Tumor Program (SKTP) database containing patients presented at Atrium Health Carolinas Medical Center's (AHCMC) Small Kidney Tumor Conference (SKTC). The conference utilizes multidisciplinary input from Urologists, Pathologists and Radiologists to evaluate renal mass patients and formulate a plan of care.

Area Deprivation Index (ADI) and income data were used as mechanisms to assess patient SES. ADI data, which combines US census block‐level data for income, education, employment and housing quality, was obtained from the University of Wisconsin School of Medicine. We converted patients' self‐reported addresses into FIPS block codes and matched them to ADI block groups to determine ADI national rank. Income data was collected from the US Census Bureau American Community Survey (ACS). Median household income was assigned using FIPS block codes. We collected patient insurance status around the time of initial SKTC presentation. Insurance status could only be obtained for patients presented after 2013. Patients were grouped into three categories: Commercial (those with any private or military coverage), Medicare/Medicaid (those with only Medicare, only Medicaid or both) and uninsured (those without any coverage).

### 2.2 Patient population

Patients diagnosed with a primary SRM and presented at SKTC from November 2009 to March 2020 were included. Patients with treatment dates following March 1, 2020, were excluded to account for possible delays due to the COVID‐19 pandemic. Only patients with masses ≤ 4 cm at initial clinic presentation and who received treatments of cryoablation, partial nephrectomy (PN) and radical nephrectomy (RN) were included. Patients that underwent short‐term active surveillance (AS) prior to treatment or experienced a delay in treatment due to additional imaging or personal preference were excluded because it is difficult to assign a definitive start date for AS, despite it being a successful treatment technique for SRMs. Race/ethnicity was determined by patient self‐report. Racial/ethnic groups were initially divided as non‐Hispanic White, Hispanic, Black, Asian, American Indian/Alaska Native and Other, but Hispanic, Asian, American Indian/Alaska Native and Other were combined for statistical analysis, given small sample sizes.

### 2.3 Statistical analysis

The primary endpoint was the time to treatment initiation (TTI‐OV), calculated as the number of days from the initial office visit for the SRM to the procedure date. We also investigated the secondary metric TTI‐Imaging, calculated as the number of days from the first time the SRM was seen on imaging to the procedure date. Demographic and clinical characteristics of patients were reported and compared based on race/ethnicity using descriptive statistics. For continuous variables, means and standard deviations (SD) were reported, while categorical variables were reported as frequencies and percentages. Corresponding P values were calculated using Fisher's exact tests for categorical variables and the Wilcoxon rank sum tests for continuous variables. We performed analyses using Kaplan–Meier, univariate and multivariable Cox Proportional Hazard models to evaluate the effect of race, ethnicity, ADI, income, procedure year, insurance status, procedure type, Charleston Comorbidity Index (CCI) and mass size at first presentation at clinic on TTI‐OV, and TTI‐Imaging. All Cox Proportional Hazard model analyses were performed via a stepwise model selection procedure, where all covariates were first included in the univariate analysis, and covariates with P‐value < 0.1 were entered into the multivariable model and only covariates with P‐value < 0.1 were retained in the final model. All statistical tests were two‐sided, and a significance level of P < 0.05 was used to determine statistical significance. All statistical analyses were conducted using SAS 9.4.

## 3 RESULTS

### 3.1 Patient characteristics

We identified 275 patients from the SKTP database who underwent cryoablation, PN or RN for a SRM from November 2009 through March 1, 2020. Of the total population, 58.18% were male and 70.18% were White, with a median age of 61 years. A total of 13.82% underwent cryoablation, 66.91% underwent PN and 19.27% underwent RN. Additional demographic and clinical characteristics are available in Table 1.

**TABLE 1:** Patient demographics and clinical characteristics by race.

	Overall	Non‐Hispanic white	Black	Other	P value
	N = 275	N = 193	N = 65	N = 17	
**Demographic Characteristics, n (%)**					
**Age, year**					<0.0001
Mean	59.00	61.00	59.00	45.00	
Median	61.00	62.00	60.00	43.00	
Q1, Q3	50.00, 69.00	52.00, 70.00	52.00, 66.00	37.00, 52.00	
Range	(20.00–89.00)	(26.00–89.00)	(34.00–82.00)	(20.00–70.00)	
**Sex**					0.8
Female	115 (42)	79 (41)	28 (43)	8 (47)	
Male	160 (58)	114 (59)	37 (57)	9 (5)	
**ADI Quartile**					0.03
Q1 = 1–25	59 (21)	44 (23)	9 (14)	6 (35)	
Q2 = 26–50	61 (22)	47 (24)	11 (17)	3 (18)	
Q3 = 51–75	88 (32)	65 (34)	19 (29)	4 (24)	
Q4 = 76–100	67 (24)	37 (19)	26 (40)	4 (24)	
**ACS Economics/Median Household Income**					<0.0001
<30,000	17 (7)	10 (6)	7 (12)	0 (0)	
30 000–59 999	91 (36)	48 (28)	34 (57)	9 (53)	
60 000–89 999	67 (27)	52 (30)	14 (23)	1 (6)	
90 000–119 999	44 (18)	36 (21)	4 (7)	4 (24)	
≥ 120 000	31 (12)	27 (16)	1 (2)	3 (18)	
**Procedure Year**					0.03
2009–2010	20 (7)	15 (8)	4 (6)	1 (6)	
2011	24 (9)	20 (10)	4 (6)	0 (0)	
2012	27 (10)	21 (11)	5 (8)	1 (6)	
2013	37 (13)	26 (13)	7 (11)	4 (24)	
2014	42 (15)	30 (16)	10 (15)	2 (12)	
2015	50 (18)	41 (21)	7 (11)	2 (12)	
2016	31 (11)	21 (11)	9 (14)	1 (6)	
2017	30 (11)	13 (7)	14 (22)	3 (18)	
2018–2020	14 (5)	6 (3)	5 (8)	3 (18)	
**Insurance Status**					<0.0001
Commercial	106 (68)	82 (80)	20 (46)	4 (40)	
Medicare/Medicaid	45 (29)	20 (20)	21 (49)	4 (40)	
Uninsured	4 (2)	0 (0)	2 (5)	2 (20)	
**Age Adjusted CCI**					0.001
Mean	1.8	1.8	2.4	0.41	
Median	0.00	0.00	3.00	0.00	
Q1, Q3	0.00, 4.00	0.00, 3.00	0.00, 4.00	0.00, 0.00	
Range	(0.00–9.00)	(0.00–9.00)	(0.00–7.00)	(0.00–3.00)	
**Clinical Characteristics, n (%)**					
**Procedure Type, n (%)**					<0.0001
Cryo	38 (14)	28 (14)	9 (14)	1 (6)	
PN	184 (67)	137 (71)	31 (48)	16 (94)	
RN	53 (19)	28 (14)	25 (38)	0 (0.00)	
**Mass size at first presentation at clinic**					0.3
Mean	2.50	2.54	2.39	2.40	
Median	2.50	2.50	2.30	2.30	
Q1, Q3	2.00, 3.00	2.00, 3.00	2.00, 2.70	1.50, 3.20	
Range	(0.2–4.0)	(0.2–4.0)	(1.0–4.0)	(1.1–4.0)	
**Tumour Size when first seen on imaging**					0.06
Mean	2.30	2.40	2.20	2.20	
Median	2.40	2.50	2.20	2.20	
Q1, Q3	1.70, 2.95	1.80, 3.00	1.70, 2.60	1.35, 3.00	
Range	(0.05–4.60)	(0.20–4.00)	(0.05–4.60)	(1.10–3.80)	

*P*‐values were determined using Fisher's exact test for categorical variables and Wilcoxon rank sum test for continuous variables.

Race/ethnicity was associated with age, CCI and procedure type. Notably, racial groups differed significantly in ADI (p = 0.03), with a larger proportion of Black patients in Q4, and in income (p < 0.0001), with more than half of all Black and Other patients earning 30,000–59,999. Racial groups also differed significantly in insurance status (p < 0.0001), with greater proportions of minority Medicare/Medicaid patients, and across procedure year as our cohort grew more diverse over time (p = 0.03) (Table 1).

Among the cohort, pathology results were available for most patients. Benign tumours accounted for 22% of the patient cohort (n = 60), whereas malignant tumours accounted for 78% of patients (n = 214). Of the malignant tumours with available grading (n = 183), low‐grade (Grade 1 or 2) accounted for 73% of cases (n = 133) and high‐grade (Grade 3 or 4) accounted for 27% (n = 50).

### 3.2 TTI‐OV

Mean TTI‐OV intervals were 82, 109 and 60 days for White, Black and Other patients, respectively (Table 2). Using the Cox Proportional Hazard model, we identified a significant effect of race/ethnicity on TTI‐OV. Black patients experienced significantly longer TTI‐OV intervals when compared to White patients (HR = 0.64, 95% CI [0.48–0.85], p = 0.005). We also identified a significant effect of procedure year on TTI‐OV. Patients treated in the second half of our cohort experienced significantly longer TTI‐OV intervals compared to earlier patients (2009–2010 vs 2018–2020: HR = 3.9, 95% CI [1.9–7.9], p = 0.0004). However, there was no significant effect of ADI, income, insurance status, procedure type, CCI or mass size at the initial office visit on TTI‐OV (Table 3).

**TABLE 2:** Time to treatment initiation in days overall and by race.

	Overall	Non‐Hispanic white	Black	Other
	N = 275	N = 193	N = 65	N = 17
**Time to Treatment Initiation (TTI‐OV), Days**				
Mean	87.00	82.00	109.00	60.00
Median	59.00	54.00	85.00	57.00
Q1, Q3	34.0, 94.0	29.50, 85.00	62.00, 115.00	34.00, 84.00
Range	(3.00–1485.00)	(3.00–1485.00)	(13.00–545.00)	(8.00–118.00)
**Time to Treatment Initiation (TTI‐Imaging), Days**				
Mean	307.00	217.00	585.00	265.00
Median	111.00	97.00	144.00	132.00
Q1, Q3	69.00, 254.00	63.00, 190.00	106.00, 502.00	66.00, 172.00
Range	(11.00–4445.00)	(11.00–2131.00)	(21.00–4445.00)	(34.00–2066.00)

We reported the mean, median and interquartile ranges (IQR) for continuous variables.

**TABLE 3:** Cox proportional hazards model (univariable and multivariable) for time to treatment initiation (TTI‐OV) and time to treatment imaging (TTI‐imaging).

Primary outcome: time to treatment initiation (TTI‐OV), days					Outcome: time to treatment imaging (TTI‐imaging), days			
Variable	Univariable analysis		Multivariable analysis		Univariable analysis		Multivariable analysis	
	Hazard ratio (95% CI)	*P*‐value	Hazard ratio (95% CI)	*P‐*value	Hazard ratio (95% CI)	*P*‐value	Hazard ratio (95% CI)	*P‐*value
**Race/Ethnicity**		0.005		0.01		0.0002		0.2
Black vs Non‐Hispanic White	0.637 (0.479–0.848)		0.698 (0.519–0.938)		0.541 (0.402–0.727)		0.734 (0.529–1.017)	
Other vs Non‐Hispanic White	1.2 (0.71–1.9)		1.4 (0.85–2.4)		0.89 (0.54–1.5)		0.92 (0.53–1.6)	
**ADI Quartile**		0.89				0.71		
Q1 vs Q2	1.0 (0.70–1.4)				1.2 (0.86–1.8)			
Q3 vs Q2	1.1 (0.76–1.5)				1.1 (0.78–1.5)			
Q4 vs Q2	0.92 (0.65–1.3)				1.1 (0.78–1.6)			
**ACS Economics/Median Household Income**		0.99				0.93		
<30,000 vs 30,000‐59,999	0.98 (0.58‐1.6)				0.86(0.51‐ 1.4)			
60 000–89 999 vs 30 000–59 999	0.99 (0.73–1.4)				0.90 (0.66–1.2)			
90 000–119 999 vs 30 000–59 999	1.0 (0.70–1.5)				0.89 (0.62–1.3)			
≥ 120 000–119 999 vs 30 000–59 999	1.1 (0.74–1.7)				1.0 (0.67–1.5)			
**Procedure Year**		0.0004		0.0010		0.0001		0.002
2009–2010 vs 2018–2020	3.9 (1.9–7.9)		3.9 (1.9–8.0)		3.1 (1.6–6.3)		2.8 (1.3–6.0)	
2011 vs 2018–2020	2.9 (1.4–5.7)		2.8 (1.4–5.7)		2.0 (1.0–3.8)		1.2 (0.59–2.5)	
2012 vs 2018–2020	2.9 (1.5–5.8)		3.0 (1.5–5.9)		1.9 (0.98–3.6)		1.9 (0.95–3.8)	
2013 vs 2018–2020	2.3 (1.2–4.5)		2.3 (1.2–4.4)		2.4 (1.3–4.4)		1.5 (0.76–2.9)	
2014 vs 2018–2020	2.5 (1.3–4.7)		2.6 (1.4–5.0)		2.7 (1.5–5.0)		2.3 (1.2–4.4)	
2015 vs 2018–2020	1.8 (0.99–3.4)		1.8 (0.98–3.4)		1.8 (0.98–3.2)		1.5 (0.80–2.8)	
2016 vs 2018–2020	1.7 (0.86–3.2)		1.7 (0.88–3.3)		1.6 (0.82–2.9)		1.3 (0.69–2.6)	
2017 vs 2018–2020	1.4 (0.71–2.6)		1.5 (0.78–2.9)		0.97 (0.51–1.8)		0.89 (0.45–1.8)	
**Insurance Status**		0.74				0.051		
Medicare/Medicaid vs Commercial	0.90 (0.63–1.3)				0.99 (0.37–2.7)			
Uninsured vs Commercial	0.72 (0.23–2.3)				0.640 (0.23–1.8)			
**Procedure Type**		0.13				0.0013		0.19
PN vs RN	1.1 (0.76–1.6				1.8 (1.3–2.6)		1.4 (0.98–1.98)	
RN vs Cryo	0.79 (0.52–1.2)				1.5 (0.96–2.3)		1.2 (0.79–2.0)	
**CCI (Charleston Comorbidity Index)**	0.96 (0.91–1.0)	0.2			0.92 (0.87–0.98)	0.005	0.91 (0.86–0.97)	0.0030
**Mass size at first presentation at clinic/on imaging**	1.1 (0.93–1.3)	0.2			1.4 (1.2–1.6)	<0.0001	1.4 (1.2–1.6)	<0.0001

**FIGURE 1:**
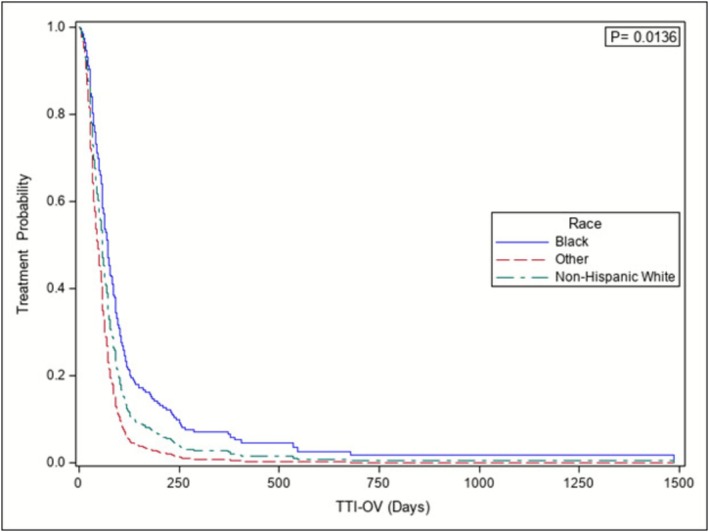
Adjusted Cox proportional hazard model‐based Kaplan–Meier curve stratified by race for TTI‐OV. Black patients had significantly longer intervals from initial office visit to treatment with adjustment for procedure year (p = 0.0136).

Upon multivariable Cox Proportional Hazard model analysis adjusting for procedure year, Black patients experienced significantly longer TTI‐OV intervals compared to White patients (HR = 0.70, 95% CI [0.52–0.94], p = 0.01) (Table 3). Adjusted Cox Proportional Hazard model‐based Kaplan Meier curve also demonstrated that Black patients experienced longer TTI‐OV intervals compared to White patients, with adjustment for procedure year (p = 0.01) (Figure 1, Figure S1).

### 3.3 TTI‐imaging

Mean TTI‐Imaging intervals were 217, 585 and 265 days for White, Black and Other patients, respectively (Table 2). Using the univariate Cox Proportional Hazard model, we found a significant effect of race/ethnicity on TTI‐Imaging with Black patients experiencing a longer interval compared to White patients (HR = 0.54, 95% CI [0.40–0.73], p = 0.0002). We again found no significant effect of ADI, income or insurance status on TTI‐Imaging. There was a significant effect of procedure year on TTI‐Imaging with quicker time to treatment in earlier years (2009–2010 vs 2018–2020: HR = 3.1, 95% CI: 1.6–6.3, p = 0.0001). Procedure type significantly impacted TTI‐Imaging, with PN scheduled more quickly than RN (HR = 1.8, 95% CI [1.3–2.6], p = 0.001). Additionally, both CCI and mass size when initially seen on imaging had a significant effect on TTI‐Imaging. For every one unit increase in CCI, patients experienced a prolonged TTI‐Imaging interval (HR = 0.92, 95% CI [0.87–0.98], p = 0.004). Also, for every one unit increase in mass size when first seen on imaging, patients experienced a quicker TTI‐Imaging interval (HR = 1.4, 95% CI [1.2–1.6], p < 0.0001) (Table 3).

**FIGURE 2:**
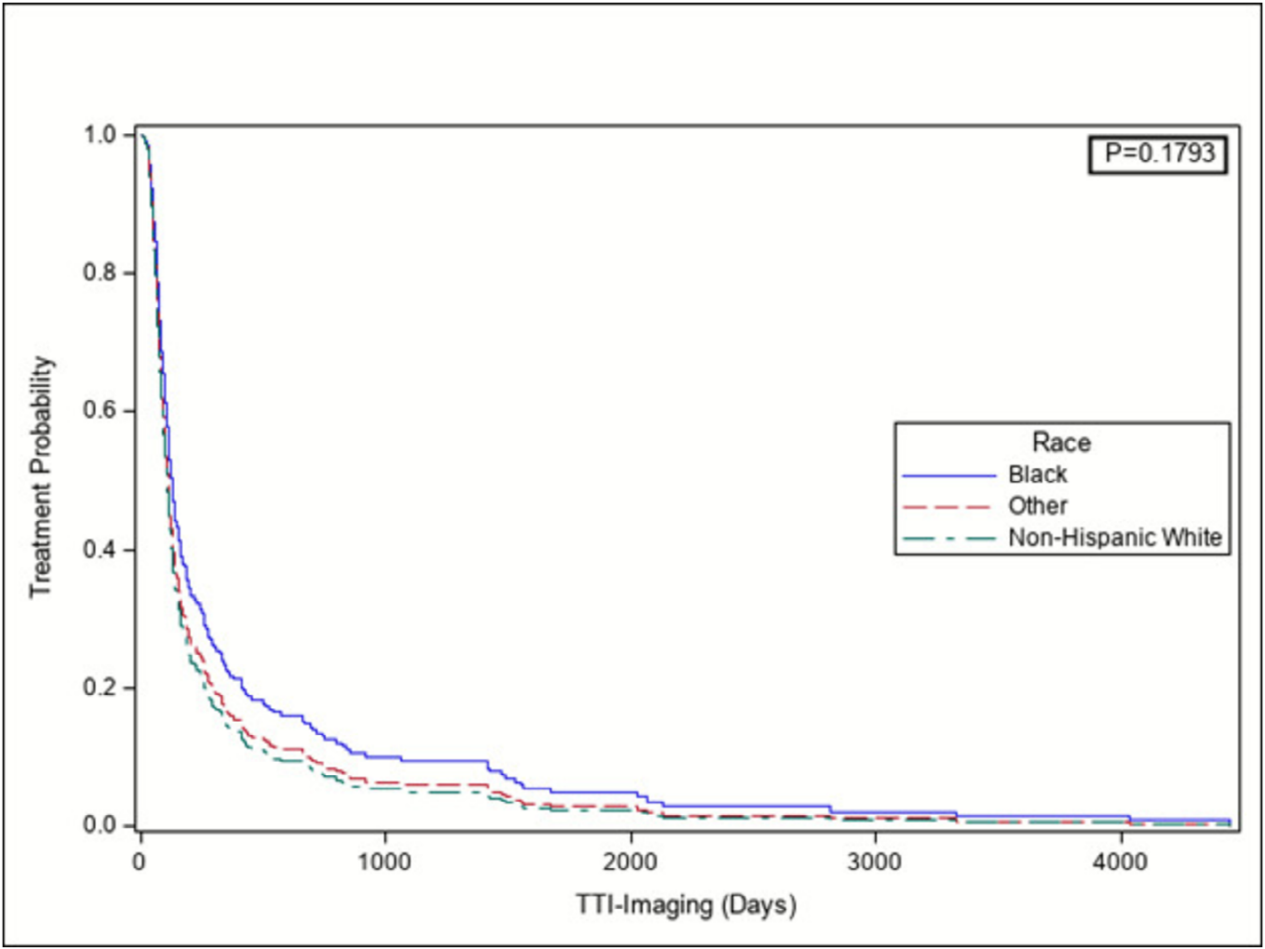
Adjusted Cox proportional hazard model‐based Kaplan–Meier curve stratified by race for TTI‐imaging. Black patients appeared to have longer intervals from when the mass was first seen on imaging to treatment, with adjustment for procedure year, procedure type, CCI, and mass size when first seen on imaging, although this was not significant (p = 0.1793).

Upon multivariable Cox Proportional Hazard model analysis adjusting for procedure type, procedure year, CCI and mass size when first seen on imaging, there was no longer a significant effect of race/ethnicity on TTI‐Imaging (p = 0.2). There was also no longer a significant effect of procedure type on TTI‐Imaging (PN vs RN HR = 1.4, CI [0.98–1.98], p = 0.2). However, a significant effect of procedure year, CCI and mass size when first seen on imaging on TTI‐Imaging was still apparent (Table 3). Adjusted Cox Proportional Hazard model‐based Kaplan Meier curve demonstrated that Black patients appear to experience longer TTI‐Imaging intervals compared to White patients with adjustment for procedure year, procedure type, CCI and mass size, although this was not significant (p = 0.2) (Figure 2, Figure S2).

## 4 DISCUSSION

This study presents novel findings on an under‐investigated health disparity, TTI, in a SRM patient population. We identified that Black race was associated with treatment delay (TTI‐OV and TTI‐Imaging). Surprisingly, lower SES as indicated by ADI, census income and insurance status, was not associated with treatment delay (TTI‐OV or TTI‐Imaging). While we observed an association between CCI and tumour size with TTI‐Imaging, we found no such association with TTI‐OV. We hypothesize this could be because TTI‐Imaging may be more significantly associated with an individual and their ability to schedule appropriate follow‐up testing. However, our TTI‐Imaging outcome could be subject to many other confounding variables, given that for a portion of the interval, patients were not under our care.

Procedure year at our medical centre was significant in relation to TTI intervals. Earlier years in our cohort (2009–2014) were associated with quicker TTI intervals compared to later years (2015–2020). Given that our centre and the population of the surrounding area grew substantially across this time period, this relationship is to be expected. However, given that patients with procedures in 2009–2010 underwent treatment over 3 times faster than patients in 2018–2020, clinics must be aware of the impact of congested provider schedules on treatment timelines. Additionally, while we initially identified that procedure type significantly impacted TTI‐Imaging, with PN scheduled more quickly than RN, upon stepwise multivariable modelling, we found no significant association. We anticipated that cryoablation may be associated with shorter treatment initiation, given that our radiology department tends to have more availability than surgery. However, we saw no such association, demonstrating that procedure type is not a hindrance for treatment at our centre.

Individual patient characteristics also appeared to be related to TTI. Patients with a higher CCI experienced a longer interval between the time the SRM was first seen on imaging and treatment. In previous studies, CCI has been associated with complication rates in RN patients and surgical delay in spinal cord injury patients.^12^, ^13^ Our study corroborates these findings, identifying that comorbid patients experience longer TTI intervals. These time‐to‐treatment differences may be due to surgical clearances or treatment accommodations that prolong the preoperative time period. Patients with a larger mass size when first seen on imaging experienced a quicker time to treatment. Given that the probability of metastatic disease increases as SRM size increases, these results demonstrate that our centre may prioritize treatment scheduling for patients with the most concerning SRMs.^14^

Pathology data further contextualizes these findings. The presence of both benign and low‐grade malignant tumours in the cohort suggests that not all SRMs carry the same urgency for intervention. This variability in tumour biology may influence clinical decision‐making and prioritization, potentially interacting with patient‐level factors such as race or comorbidity. Future work should explore whether disparities in TTI are more pronounced among patients with aggressive pathology, where delays may have greater clinical consequences.

Our results echo results from previous studies that identified racial disparities in TTI in urology. Prior research indicated that Black prostate cancer as well as Black and Hispanic muscle invasive bladder cancer patients experience longer intervals from diagnosis to start of treatment compared to White patients.^9^, ^15^, ^16^ Additionally, previous work has shown that Black patients were significantly more likely to defer therapy than their White counterparts, and experience worse overall survival, even with comparable cancer‐specific outcomes.^17^ While racial disparities in urology have been widely investigated, few studies have explored time disparities in kidney cancer patients. One study investigating preoperative surgical wait time disparities in RCC patients found that Hispanic Americans experience longer surgical wait times.^18^ Our study on SRM patients similarly found that race is associated with treatment delays, with Black patients experiencing longer treatment waiting periods. These racial disparities do not appear to be explained by SES status, as ADI, income and insurance status had no association with TTI‐OV or TTI‐Imaging.

Our study does have several limitations. We chose to exclude patients on active surveillance, which could result in a potentially higher‐risk cohort. SES status is subject to change over time, and our income, ADI and insurance data were obtained for one specific time. Additionally, given our single‐institution patient cohort, the generalizability of our findings may be limited. We also had small sample sizes for racial groups other than Black and White, resulting in our need to combine these groups for analysis purposes.

## 5 CONCLUSION

Our study demonstrates that Black race and procedure years later in our cohort were associated with longer TTI‐OV intervals. Later procedure years, higher CCI and smaller mass size when first identified on imaging were associated with longer TTI‐Imaging intervals. Lower SES, as indicated by the ADI, census income and insurance status, was not associated with TTI.

## AUTHOR CONTRIBUTIONS

All authors listed above have contributed to the work herein as outlined by ICMJE criteria and agree to their inclusion in this manuscript.

## CONFLICT OF INTEREST STATEMENT

The authors declare no conflict of interest.

## ETHICS APPROVAL

This is a retrospective observational study of a prospectively managed database. The study has gone through the Atrium Health IRB Committee and has been approved (IRB #00091422). All procedures were in accordance with the 1964 Helsinki Declaration and ethical standards of the responsible committee on human experimentation (institutional and national).

## Supporting information


**Figure S1.** Kaplan–Meier curve stratified by race for TTI‐OV. Black patients had significantly longer intervals from initial office visit to treatment (p = 0.0044).

**Figure S2.** Kaplan–Meier curve stratified by race for TTI‐Imaging. Black patients appeared to have longer intervals from when the mass was first seen on imaging to treatment (p = 0.0002).

## Data Availability

The datasets generated during and/or analysed during the current study are available from the corresponding author on reasonable request.
