# Supplementary figures and images for: Racial and socioeconomic disparities from time of diagnosis to treatment for small renal masses
Source: BJUI Compass. 2026 Feb 3;7(2):e70115. doi: 10.1002/bco2.70115 (PMC12868985; doi:10.1002/bco2.70115)

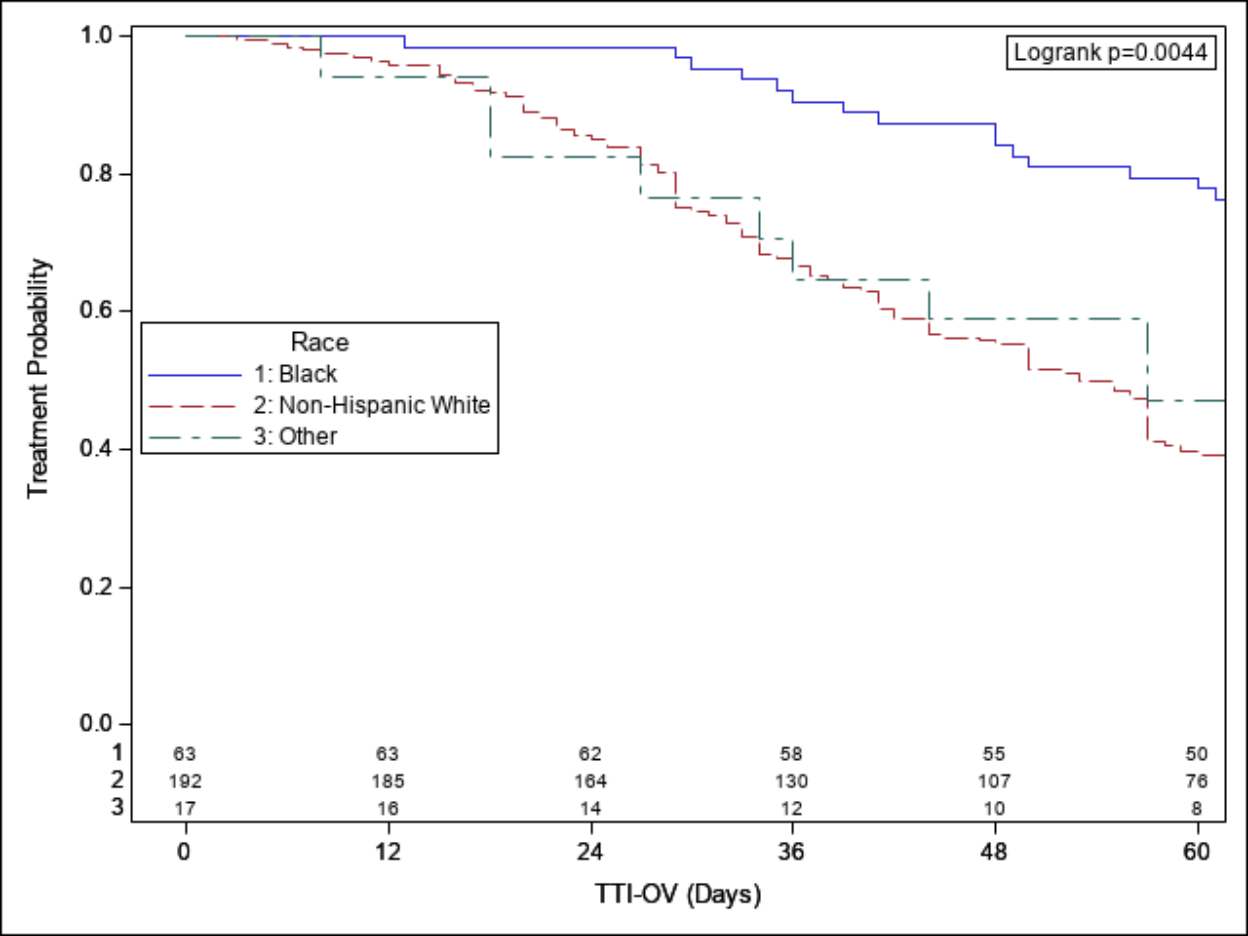

Supplement: Supplementary file 1 — Figure S1. Kaplan–Meier curve stratified by race for TTI‐OV. Black patients had significantly longer intervals from initial office visit to treatment (p = 0.0044). [file BCO2-7-e70115-s001.png]

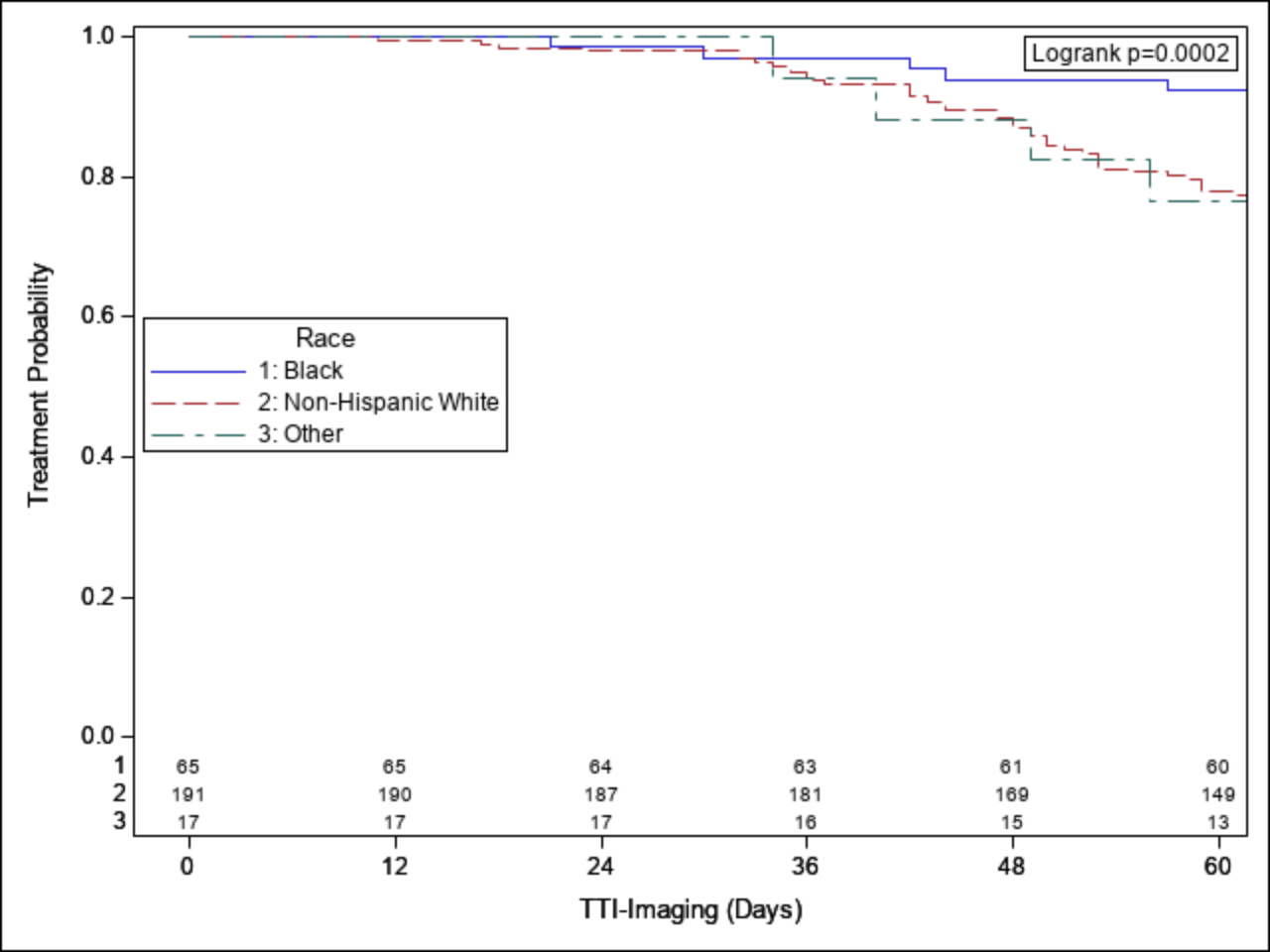

Supplement: Supplementary file 2 — Figure S2. Kaplan–Meier curve stratified by race for TTI‐Imaging. Black patients appeared to have longer intervals from when the mass was first seen on imaging to treatment (p = 0.0002). [file BCO2-7-e70115-s002.png]
